# Supplementary material for: Can Essential Oils Be a Natural Alternative for the Control of Spodoptera frugiperda? A Review of Toxicity Methods and Their Modes of Action
Source: Plants (Basel). 2022 Dec 20;12(1):3. doi: 10.3390/plants12010003 (PMC9823514; doi:10.3390/plants12010003)
Supplement: Supplementary file 1 [file plants-12-00003-s001.zip › plants-2024874-supplementary Material S1.pdf]

Supplementary Material S1

Table S1 *Spodoptera* pest species on which the toxicity of EOs was evaluated

Spodoptera species	Occurrence in the literature
S. littoralis	109
S. litura	81
S. exigua	18

Table S2 Methods used in *S. littoralis, S. litura* and *S. exigua* to assess the toxicity of EOs

Toxicity methods	Occurrence in the literature
Topical application	123
Fumigant	47
Toxicity by ingestion	41
nd	1

nd: non determined.

Table S3 Times at which the mortality of S. *littoralis, S. litura* and *S. exigua* was recorded.

Time	Occurrence in the literature
24 h	146
72 h	27
48 h	17
96 h	4
nd	3
12 h	1
120 h	1
168 h	1

nd: non determined.

Table S4 Larval stages of S. *littoralis, S. litura* and *S. exigua* used to assess the toxicity of EOs.

Larval stage	Occurrence in the literature
3	175
2	20
4	10
1	1
5	1

Table S5 Families whose essential oil has been studied as an insecticide against *S. littoralis, S. litura* and *S. exigua*.

Plant family	Occurrence in the literature
Lamiaceae	89
Apiaceae	30
Zingiberaceae	19
Rutaceae	16
Araceae	11
Cupressaceae	9
Myrtaceae	9
Asteraceae	9
Verbenaceae	6
Pinaceae	6
Lauraceae	5
Poaceae	4
Annonaceae	3
Myoporaceae	2
Illiciaceae	1
Valerianaceae	1
Geraneacea	1
Cardiopteridaceae	1
Burseraceae	1
Santalaceae	1
Cannabaceae	1
Amaryllidaceae	1

Table S6 Plant species whose Essential oils are evaluated as insecticides against *S. littoralis, S. litura* and *S. exigua*.

Plant specie	Occurrence in the literature
Foeniculum vulgare	13
Acorus calamus	11
Crithmum maritimum	8
Curcuma longa	7
Salvia officinalis	7
Alpinia pyramidata	6
Coleus amboinicus	6
Lantana camara	6
Lavandula latifolia	5
Origanum majorana	5
Pelargonium graveolens	5
Lavandula angustifolia	4
Mentha arvensis	4
Ocimum basilicum	4
Amyris balsamifera	3
Artemisia absinthium	3
Juniperus virginiana	3
Melissa officinalis	3

Mentha spicata	3
Pogostemon cablin	3
Rosmarinus officinalis	3
Zingiber officinale	3
Abies siberica	2
Citrus aurantifolia	2
Citrus limonum	2
Cymbopogon winterianus	2
Eucalyptus globulus	2
Eugenia caryophyllata	2
Juniperus communis	2
Melaleuca quinquenervia	2
Mentha citrata	2
Mentha pulegium	2
Myoporum bontioides	2
Nepeta cataria	2
Origanum compactum	2
Origanum vulgare	2
Pelargonium roseum	2
Salvia sclarea	2
Spheranthus amaranthroids	2
Tagetes bipinata	2
Thuja occidentalis	2
Thymus mastichina	2
Thymus vulgaris	2
Tsuga canadensis	2
Aframomum citratum	1
Allium sativum	1
Alpinia galanga	1
Angelica archangelica	1
Artemisia nakaii	1
Basil[a]	1
Bergamot[a]	1
Cannabis sativa	1
Chloroxylon swietenia	1
Cinnamomum verum	1
Cinnamomum zeylanicum	1
Cinnamon[a]	1
Citronella[a]	1
Citrus aurantium	1
Citrus bergamia	1
Citrus limon	1
Clary sage[a]	1
Clove bud[a]	1
Coriandrum sativum	1
Cuminum cyminum	1
Cymbopogon nardus	1
Cupressus sempervirens	1

Cypress[a]	1
Daucus carota	1
Eucalyptus radiata	1
Fennel[a]	1
Fennel sweet[a]	1
Frankincense[a]	1
Geranium[a]	1
Hedychium coccineum	1
Helosciadium nodiflorum	1
Illicium verum	1
Lavander (Bulgariam) [a]	1
Lavander (french) [a]	1
Lemon[a]	1
Lemongrass[a]	1
Majorana hortensis	1
Mandarin[a]	1
Marjoram[a]	1
Mentha aquatica	1
Mentha longifolia	1
Mentha x piperita	1
Monodora myristica	1
Ocimum tenuiflorum	1
Ocimum gratissimum	1
Orange sweet[a]	1
Origanum creticum	1
Origanum syriacum syriacum	1
Patchouli[a]	1
Peppermint[a]	1
Pimpinella anisum	1
Pine[a]	1
Ridolfia segetum	1
Rosemary[a]	1
Salvia hispanica	1
Salvia veneris	1
Sandal wood[a]	1
Spearmint[a]	1
Sweet thyme[a]	1
Thymus serpyllum	1
Valeriana jatamansi	1
Xylopia aethiopica	1
ylang ylang	1
Wedelia prostrata	1
Zanthoxylum armatum	1
Camphor essential oil[a]	1
Castor essential oil[a]	1
withe thyme	1
Hyssopus officinalis	1
Matricaria recutita	1

Syzygium aromaticum	1
Pinus mugo	1
Cinnamomum verum	1
Cinnamomum cassia	1
Satureia hortensis	1

[a]Commercial EOs. Plant species name not provided.

Table S7 Lethal effects of EOs tested by the Topical application method in 3rd larval stage of *Spodoptera litura, S. littoralis* and *S. exigua*.

Spodoptera species	Essential oil	50% Lethal Doses (LD_{50}; μg/ insect)	Reference
S. exigua	*Rosmarinus officinalis*	5.85 x 10^{-4}[*1]	[160]
	Salvia hispanica	18.4[2]	[110]
	Salvia hispanica	24.8[1]	[110]
	Salvia veneris	69[1]	[161]
	Citrus bergamia	146.17[*2]	[160]
	Citrus aurantium	276.74[*2]	[160]
	Amyris balsamifera	334.78[*1]	[160]
	Cymbopogon winterianus	533.44[*1]	[162]
S. littoralis	*Nepeta cataria*	3.6 x 10^{-5}[*1]	[162]
	Lavandula latifolia	5.4x 10^{-5}[*1]	[162]
	Mentha citrata	3.7 x 10^{-4}[*1]	[162]
	Thymus mastichina	5.13 x 10^{-4}[*1]	[162]
	Ocimum basilicum	5.85 x 10^{-4}[*1]	[162]
	Lavandula angustifolia	8.28 x 10^{-4}[*1]	[162]
	Eugenia caryophyllata	29.3[1]	[162]
	Ocimum gratissimum	30.2[1]	[163]
	Melaleuca quinquenervia	31.1[1]	[162]
	Ridolfia segetum	37.9[1]	[164]
	Pimpinella anisum	57.3[1]	[165]
	Mentha spicata	58[1]	[162]
	Mentha pulegium	63[1]	[162]
	Thymus vulgaris	71[1]	[162]
	Pogostemon cablin	78[1]	[162]
	Origanum vulgare	82[1]	[162]
	Rosmarinus officinalis	89[1]	[162]
	Salvia officinalis	93[1]	[162]
	Origanum compactum	96[1]	[162]
	Cuminum cyminum	100[1]	[165]
	Origanum syriacum	103.3[1]	[166]
	Helosciadium nodiflorum	116.7[1]	[167]
	Aframomum citratum	119.2[1]	[168]
	Cannabis sativa	152.3[1]	[169]
	Tsuga canadensis	318.16[1]	[162]
	Abies siberica	596.54[1]	[162]
S. litura	*Alpinia pyramidata*	13.26[1]	[170]
	Curcuma longa	120.4[1]	[170]
	Coleus amboinicus	123.6[1]	[170]
	Alpinia pyramidata	130.4[1]	[170]
	Curcuma longa	137.6[1]	[170]
	Alpinia pyramidata	142.5[1]	[170]
	Curcuma longa	168.6[1]	[170]
	Basil[a]	242.7[1]	[171]
	Lemongrass[a]	243.5[1]	[171]
	Spearmint[a]	243.8[1]	[171]

	Geranium[a]	247.1[1]	[171]
	Peppermint[a]	263.3[1]	[171]
	Clove bud[a]	266.2[1]	[171]
	Fennel sweet[a]	277.2[1]	[171]
	Patchouli[a]	289.1[1]	[171]
	sweet thyme[a]	307.6[1]	[171]
	Citronella[a]	398.1[1]	[171]
	Marjoram[a]	648.4[1]	[171]
	Frankincense[a]	>1000[1]	[171]
	Lavander (Bulgariam) [a]	>1000[1]	[171]
	Rosemary[a]	>1000[1]	[171]

All values marked with an asterisk (*) were recalculated for comparison.

[1]Determined at 24 hs.

[2]Determined at 48 hs.

[a]Commercial EOs. Plant species name not provided.

Table S8 *Spodoptera* pest species on which the toxicity of pure VOCs was evaluated

Spodoptera species	Occurrence in the literature
S. littoralis	58
S. litura	12
S. exigua	1

Table S9 Methods used in *S. littoralis, S. litura* and *S. exigua* to assess the toxicity of pure VOCs

Toxicity methods	Occurrence in the literature
Topical application	67
Toxicity by ingestion	6
Fumigant	2
Immersion	2
nd	1

nd: non determined.

Table S10 Times at which the mortality of S. *littoralis, S. litura* and *S. exigua* was recorded.

Time	Occurrence in the literature
24 h	66
48 h	4
nd	3
96 h	2
72 h	1

nd: non determined.

Table S11 Larval stages of S. *littoralis, S. litura* and *S. exigua* used to assess the toxicity of pure VOCs

Larval stage	Occurrence in the literature
3	48
4	25
2	1
nd	1

nd: non determined.

Table S12 Pure VOCs used to evaluate toxicity against *S. littoralis, S. litura* and *S. exigua*

Pure VOC	Occurrence in the literature
carvacrol	5
eugenol	4
limonene	4
thymol	4
β-pinene	3
(+)-camphor	3
1,8-cineole	3

γ-terpinene	3
linalool	3
p-cymene	3
(−)-camphene	3
α-terpineol	3
trans-anethole	3
β-caryophyllene	3
pogostone	3
gallic acid	2
canillin	2
α-pinene	2
(−)-borneol	2
(±)-citronellal	2
cinnamyl alcohol	2
terpinen-4-ol	2
2,6-dimethoxyphenol	1
2-ethylphenol	1
2-methoxy-4-methylphenol	1
4-allylanisole	1
4-ethylguaiacol	1
4-ethylphenol	1
β-citronellol	1
caffeic acid	1
cinnamaldehyde	1
dimethyl sulfide	1
estragole	1
ferulic acid	1
geijerene	1
geraniol	1
guaiacol	1
isoeugenol	1
L-carvone	1
menthone	1
myrcene	1
p-coumarid acid	1
phloroglucinol	1
pregeijerene	1
rotenone	1
sabinene	1
salicylaldehyde	1
salicylic acid	1
sinapic acid	1
syringic acid	1
terpinolene	1
trans-cinnamic acid	1
vanillic acid	1
α-phellandrene	1
3-carene	1

α-thujone	1
(-)-bornyl acetate	1
α-caryophyllene	1
γ-elemene	1
α-humulene	1
(E,E)-α-farnesene	1
2,6-octadienal, 3,7-dimethyl-, (Z	1
eucalyptol	1
p-menth-1-en-8-ol	1
1,6-octadien-3-ol, 3,7-dimethyl	1
1-cyclohexyl-2-buten-1-o	1
pulegone	1

Table S13 Lethal effects of pure VOCs tested, on 3rd larval stage of *Spodoptera littura, S. litoralis* and *S. exigua*, by Topical application method.

Spodoptera specie	Compound	50% Lethal Doses ($LD_{50;}$ μg/ insect)	Reference
S. littoralis	thymol	9[1]	[172]
	γ-terpinene	11.86[1]	[173]
	carvacrol	15[1]	[172]
	terpinen-4-ol	16.2[1]	[173]
	trans-anethole	18[1]	[172]
	L-carvone	18[1]	[172]
	menthone	25[1]	[172]
	geraniol	25.2[1]	[168]
	(±)-citronellal	31[1]	[172]
	β-citronellol	31[1]	[172]
	cinnamaldehyde	32[1]	[172]
	γ-terpinene	34[1]	[172]
	4-allylanisole	35[1]	[172]
	eugenol	40[1]	[172]
	α-terpineol	43[1]	[172]
	isoeugenol	44[1]	[172]
	sabinene	45.1[1]	[168]
	p-cymene	46[1]	[172]
	terpinolene	52[1]	[172]
	p-cymene	52.3[1]	[168]
	α-phellandrene	54.6[1]	[168]
	1,8-cineole	56[1]	[172]
	cinnamyl alcohol	61[1]	[172]
	(−)-β-pinene	65[1]	[172]
	(+)-camphor	71[1]	[172]
	β-pinene	84.5[1]	[168]
	linalool	85[1]	[172]
	γ-terpinene	89[1]	[172]
	myrcene	89[1]	[172]
	α-pinene	97[1]	[172]
	(R)-(+)-limonene	122[1]	[172]
	α-pinene	123.5[1]	[168]
	(−)-camphene	>300[1]	[172]
	trans-cinnamic acid	>300[1]	[172]
	vanillin	>300[1]	[172]
	gallic acid	>300[1]	[172]
	(−)-borneol	>300[1]	[172]
	dimethyl sulfide	>300[1]	[172]
S. litura	thymol	22[1]	[174]
	carvacrol	38.3[1]	[166]
	carvacrol	42.7[1]	[174]
	anethole	62[1]	[174]
	citronellal	110[1]	[174]
	terpineol	141.3[1]	[174]
	estragole	142.2[1]	[171]
	eugenol	157.6[1]	[174]
	linalool	178.6[1]	[171]

	limonene	395.6[1]	[171]
	α-thujone	37.1[2]	[110]
	α-caryophyllene	40.4[2]	[110]
	p-cymene	50.7[2]	[110]
	(R)-(+)-limonene	67.2[2]	[110]
	3-carene	77.4[2]	[110]
	(-)-bornyl acetate	82.6[2]	[110]
S. exigua	β-pinene	102.4[2]	[110]
	1,8-cineole	104.2[2]	[110]
	borneol	120[2]	[110]
	linalool	124.2[2]	[110]
	β-caryophyllene	134.8[2]	[110]
	(+)-camphor	161.2[2]	[110]
	camphene	194.6[2]	[110]

All values marked with an asterisk (*) were recalculated for comparison.
[1]Determined at 24 hs.
[2]Determined at 48 hs.
[a]Commercial EOs. Plant species name not provided.
