# Supplementary material for: Can Essential Oils Be a Natural Alternative for the Control of Spodoptera frugiperda? A Review of Toxicity Methods and Their Modes of Action
Source: Plants (Basel). 2022 Dec 20;12(1):3. doi: 10.3390/plants12010003 (PMC9823514; doi:10.3390/plants12010003)
Supplement: Supplementary file 1 [file plants-12-00003-s001.zip › plants-2024874-supplementary Material S2.pdf]

Supplementary Material S2

Table S14 Results of the bibliographic search.

Data base	Date of search	Search results*	Discarded items	Reasons for discarding
Scopus	27/5/2022	22	6	Don't use EOs.
Google Scholar	11/7/2022	2200	2189	Use only engineered compounds.<br>Work only with cell cultures.
Scielo	8/7/2022	3	2	Determine other parameters and do
ScienceDirect	8/7/2022	137	132	not determine mortality.
Total		2362		Are not research articles.<br>Repeated items

*Construct used: "*Spodoptera frugiperda*" AND "essential oils" AND "toxicity"
