# Supplementary material for: Can Essential Oils Be a Natural Alternative for the Control of Spodoptera frugiperda? A Review of Toxicity Methods and Their Modes of Action
Source: Plants (Basel). 2022 Dec 20;12(1):3. doi: 10.3390/plants12010003 (PMC9823514; doi:10.3390/plants12010003)
Supplement: Supplementary file 1 [file plants-12-00003-s001.zip › plants-2024874-supplementary Material S3.pdf]

Supplementary Material S3

Table S15 Main organic compounds of Essential oils used used as insecticides against *Spodoptera frugiperda.*

Principal compound* of EOs	Occurence in the literature (%)**
geranial	11
linalool	9
α-pinene	9
limoneno	5
(E)-anetol	4
citronelal	4
β-thujone	4
citronellol	4
α-thujone	4
nonanone	4
neral	4
safrol	4
acetophenone	4
menthone	4
3-carene	4
β-bisabolol	2
methyl-chavicol	2
bicyclogermacrene	2
1-butyl-3.4-methylenedioxybenzene	2
zingeberene	2
dillapiole	2
precoceno	2
β-myrcene	2
carvona	2
α-terpineol	2
δ-cadinene	2
1-butyl-3,4-methylenedioxybenzene	2
2-methoxy-4,5- (methylenedioxy) Propiophenone	2
exalatacin	2
(E)-caryophyllene	2

*Most abundant (quantity) compound in EO composition.
**Percentages calculated based on the analysis of the composition of the 57 EO studied.
